# A study of Candida biofilms in intensive care patients

**DOI:** 10.1186/cc11726

**Published:** 2012-11-14

**Authors:** R Kaur, R Goyal, M Singh, P Bhalla, R Kumar

**Affiliations:** 1 Maulana Azad Medical College, New Delhi, India

## Background

Advances in modern medicine and patient management have seen increased use of prosthetic materials, leading to biofilm formation, and the constituent organisms are different in their growth rates and susceptibility patterns and cause many complications. Candida species are increasingly seen to be important nosocomial pathogens and are major causes of morbidity and mortality in immunocompromised patients of intensive care and postoperative wards. Hence our aims were to isolate and identify specific Candida species associated with the luminal biofilm in endotracheal tubes/i.v. catheters in ICU patients and to study the pathogenicity of Candida species by testing for virulence mechanisms including biofilm formation and antifungal susceptibility.

## Methods

One hundred and twenty-five medical/postoperative patients admitted to the ICU for more than 48 hours were studied. Tracheal tubes at the time of extubation and intravenous/other catheters removed from patients and blood samples and other relevant samples from potentially infected sites were collected and processed. Direct microscopy done by KOH mount and Gram staining and cultures were done on blood agar, brain heart infusion agar, and Sabouraud's Dextose agar with antibiotics and were incubated at 25 and 37°C. Antifungal susceptibility and virulence testing of Candida species was done by phospholipase, proteinase estimation and adherence assay.

## Results

The patients ranged from 13 to 90 years with a male predominance seen in most ages. A total of 86.29% were admitted for less than 1 week followed by 9.68% for 1 to 2 weeks. The most common organ system involved was the GIT followed by the CNS, respiratory and multiple organ systems. In total, 356 samples including indwelling devices, blood and urine samples were processed. A total of 253 samples were sterile while fungal isolations were obtained in 103 samples with 51.4% from indwelling devices and 39.8% from urine samples. *Candida tropicalis *(40%) and *Candida albicans *(39%) were the commonest followed by *Candida glabrata *(11%), *Candida parapsilosis *(4%) and *Candida krusei*, *Candida kefyr *and *Candida sphaerica *(each 2%). Candida biofilm formation was elicited in 53 indwelling devices. A total of 58.25% Candida species were resistant to fluconazole. Phospholipase and proteinase activities were seen in 73.8% and 55.3% Candida isolates with different species showing a wide range of activities, while 71 Candida isolates showed (4+) adherence activity.

## Conclusion

Candida species continue to be important fungal pathogens in the formation of biofilms in ICU patients and knowing drug susceptibility and virulence characteristics will help in reducing associated complications, device control strategies and consequently reducing morbidity, mortality and treatment costs.

